# Vitamin D and Skeletal Muscle: Current Concepts From Preclinical Studies

**DOI:** 10.1002/jbm4.10575

**Published:** 2021-11-15

**Authors:** Christian M. Girgis, Tara C. Brennan‐Speranza

**Affiliations:** ^1^ Faculty of Medicine and Health University of Sydney Sydney NSW Australia; ^2^ Department of Diabetes and Endocrinology Westmead Hospital Sydney NSW Australia; ^3^ Department of Endocrinology Royal North Shore Hospital Sydney NSW Australia; ^4^ School of Medical Sciences University of Sydney Sydney NSW Australia; ^5^ School of Public Health University of Sydney Sydney NSW Australia

**Keywords:** MUSCLE, VITAMIN D, VITAMIN D RECEPTOR, SARCOPENIA, SATELLITE CELLS, DEVELOPMENT

## Abstract

Muscle weakness has been recognized as a hallmark feature of vitamin D deficiency for many years. Until recently, the direct biomolecular effects of vitamin D on skeletal muscle have been unclear. Although in the past, some reservations have been raised regarding the expression of the vitamin D receptor in muscle tissue, this special issue review article outlines the clear evidence from preclinical studies for not only the expression of the receptor in muscle but also the roles of vitamin D activity in muscle development, mass, and strength. Additionally, muscle may also serve as a dynamic storage site for vitamin D, and play a central role in the maintenance of circulating 25‐hydroxy vitamin D levels during periods of low sun exposure. © 2021 The Authors. *JBMR Plus* published by Wiley Periodicals LLC on behalf of American Society for Bone and Mineral Research.

## Introduction

By the 1970s it had become clear that the vitamin D status of populations was largely determined by exposure to solar UVB light driving photochemical synthesis in the skin rather than by dietary intake. Vitamin D is produced in the skin when UV photons convert 7‐dehydrocholesterol through a two‐step process into vitamin D_3_. This is followed by 25‐hydroxylation in the liver and 1α‐hydroxylation in the kidney (predominantly) to form the metabolically active 1,25‐dihydroxyvitamin D_3_ (1,25(OH)_2_D_3_). The principal role of vitamin D in the body is to signal the absorption of calcium and phosphate from the gut. Calcium absorption across the intestinal absorptive cells, the enterocytes, occurs both via active transport, facilitated by 1,25(OH)_2_D_3_, and via paracellular diffusion through tight junctions.^(^ ^1^ ^)^ It is well established that the genomic actions of 1,25(OH)_2_D_3_ in the enterocytes result in the influx of calcium through the apical calcium channel known as the transient receptor potential vanilloid type 6 (TRPV6), followed by translocation through the cell by calbindin and finally, release across the plasma membrane via the calcium adenosine triphosphatase (ATPase) pump. The passive diffusion of calcium is less reliable and has traditionally been considered a function of the electrochemical gradient across the lumen. Increasingly, however, evidence also suggests that 1,25(OH)_2_D_3_ plays a role in improving the ion permeability of the tight junctions, and thus this passive diffusion of calcium as well.^(^ ^2^, ^3^ ^)^ Vitamin D likewise improves phosphate absorption.^(^ ^1^ ^)^ Vitamin D also increases renal calcium reabsorption resulting in a net increase in serum calcium levels. Overall, vitamin D is necessary for the maintenance of mineral homeostasis and is vital for the health of the musculoskeletal system.^(^ ^4^ ^)^

**Fig 1:**
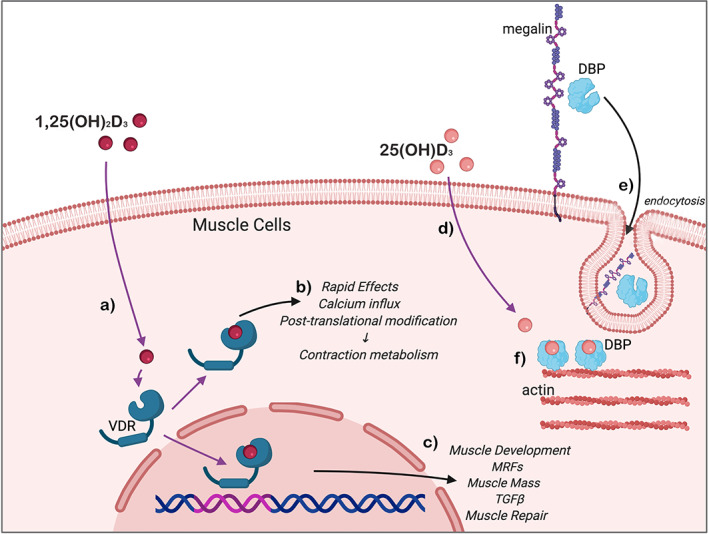
(*a*) Active 1,25(OH)_2_D_3_ diffuses across the muscle cell membrane and binds the intracellular VDR. This ligand‐receptor complex then drives either (*b*) rapid, nongenomic effects on intracellular calcium signaling pathways; or (*c*) transcriptional activity of genes involved in the differentiation of myocytes into mature contractile myotubes, maintenance of skeletal muscle quality, and protection from muscle atrophy. (*d*) Circulating 25(OH)D_3_ diffuses across the muscle cell membrane, while (*e*) VDBP requires endocytosis via megalin activity at the cell surface. (*f*) VDBP attaches to a binding site on intracellular actin filaments and binds the 25(OH)D_3_, aiding in the maintenance of circulating levels of this metabolite during winter. VDBP = vitamin D binding protein; VDR = vitamin D receptor.

The role of vitamin D in bone health has been well established for many years, although it has more recently come to light that there may also be a strong link between vitamin D and skeletal muscle health that lies outside of the circulating levels of phosphate and calcium.^(^ ^5^, ^6^, ^7^ ^)^ There are still many questions regarding the direct actions of 1,25(OH)_2_D_3_ in muscle. Severe vitamin D deficiency results in muscle weakness and vitamin D status is a predictor of muscle strength and performance in older adults,^(^ ^8^ ^)^ but whether these observations are linked to the knock‐on effects on circulating calcium and phosphate levels alone or whether vitamin D directly modulates these parameters of muscle is still hotly debated.^(^ ^9^ ^)^ This review focuses on the current literature that indicates an independent role for vitamin D in the maintenance of skeletal muscle quality, the central role of the vitamin D receptor (VDR) in muscle function as well as a summary of new research that posits the muscle as an important storage site for vitamin D and its binding protein, VDBP, to aid in the maintenance of circulating levels during winter. The various functions of vitamin D in skeletal muscle cells have been summarized in Fig. 1.

## VDR Expression in Skeletal Muscle

Following the discovery of VDR in 1974,^(^ ^10^ ^)^ our knowledge of its structural composition, biological activity, and ligand interactions have expanded considerably.^(^ ^11^ ^)^ The late Anthony W. Norman shed immense light on the complexity of the VDR, defining its protein composition through X‐ray crystallography and expounding key conformational changes that determine genomic versus rapid effects of VDR on cellular activity.^(^ ^12^ ^)^ An appreciation of structure–function interactions of VDR, and its activity as an orphan nuclear receptor, in addition to established effects through ligand binding (1,25D‐VDR‐retinoid X receptor [RXR]), provide strong mechanistic bases for vitamin D's expanding nonclassical effects beyond bone and mineral homeostasis.^(^ ^12^ ^)^

The VDR is present in invertebrates, fish, birds, and mammals, with a wide repertoire of expression in different organs and tissues. Until recently, however, the presence of VDR in skeletal muscle was unclear. There had been conflicting reports on VDR detection by Western blot and immunohistochemistry in murine and adult human muscle.^(^ ^13^, ^14^, ^15^ ^)^ Using a specific VDR antibody, VDR was found to be present at low levels^(^ ^16^ ^)^ or absent.^(^ ^15^, ^17^ ^)^ Relative to classical sites of VDR activity, such as the duodenum, its expression in muscle is substantially lower. Low baseline levels, however, do not preclude a biological role for VDR in muscle. Transcription factors may exert genomic effects even at low levels of expression, dependent on their binding affinity to DNA.^(^ ^18^ ^)^

Approaches that examine systems‐wide VDR distribution, such as the use of a luciferase‐expressing VDR transgene in a murine model,^(^ ^19^ ^)^ miss the relatively low level of expression in skeletal muscle. Protein detection methods may also not be sensitive enough to detect low baseline levels of VDR, and induction by key physiologic stimuli may be necessary for detection. In support of this, priming VDR by treatment with its ligand increased expression levels in older human subjects.^(^ ^20^, ^21^ ^)^ In another study, muscle injury relating to severe knee osteoarthritis (OA) was associated with higher VDR levels in quadriceps,^(^ ^22^ ^)^ although the VDR was clearly detected in muscles from both OA and control subjects.

VDR expression within muscle is also time‐dependent and modulated throughout muscle development and aging. In vitro, muscle precursor cells display substantially greater levels of VDR compared to fully differentiated myotubes and whole muscle fibers.^(^ ^16^, ^17^ ^)^ This is consistent with the early expression of VDR in the mesoderm, the embryonic tissue that gives rise to the musculoskeletal system.^(^ ^23^ ^)^ Mice show similar age‐dependent changes in muscle VDR with significantly higher levels in newborn mice compared to 3‐week‐old mice and adult mice.^(^ ^16^ ^)^ Muscle injury in adult mice leads to a recapitulation of embryonic myogenesis, and VDR is increased under these circumstances.^(^ ^24^, ^25^ ^)^ Interestingly, VDR is specifically expressed in satellite cells, muscle stem cells, and in vitro; 1,25(OH)D_3_ modulates myogenic cell differentiation in these primordial cells.^(^ ^26^ ^)^ Therefore, VDR's predominant expression in muscle within primordial cells, newborn mice, and regenerating muscle fibers supports a pleiotropic role of vitamin D in muscle development and repair.

In summary, a number of experimental and biological issues have confounded the clear demonstration of VDR in muscle. These include wide differences in muscle models used, nonspecificity of VDR antibodies, and protein detection methods that are insufficiently sensitive to detect low baseline levels of biologically active VDR, although most of these obstacles have now been overcome. Muscle VDR may also sequester within a specific cell population, such as satellite cells, and thus evade detection by methods examining whole tissue (satellite cells comprise ~5% cells in adult muscle).^(^ ^27^ ^)^ The weight of evidence indicates that VDR is indeed expressed in muscle but at very low baseline levels in adults, and that injury upregulates its expression. VDR in muscle predominates in precursor cells and in developing and regenerating muscle fibers. Thus, its activity in this tissue appears related to muscle development and pleiotropy.

## Vitamin D and Muscle Cells: In Vitro Models

In vitro studies using isolated myotubes and cultured muscle cell lines are one way of testing whether vitamin D and its metabolites have direct effects in muscle, bypassing the modulation of circulating calcium and phosphate mineral levels and the subsequent effects these changes may elicit in muscle. Various studies have investigated the direct effects of vitamin D using cell culture models, the majority of which have analyzed myotube formation and size,^(^ ^9^, ^26^, ^28^, ^29^, ^30^, ^31^ ^)^ the expression of proteins involved in muscle formation and function, insulin sensitivity,^(^ ^32^, ^33^, ^34^, ^35^ ^)^ glucose^(^ ^35^ ^)^ and lipid uptake and metabolism,^(^ ^36^, ^37^ ^)^ as well as mitochondrial activity.^(^ ^36^ ^)^

Most of the cell studies have employed similar methods that include incubating either isolated mouse or human skeletal myoblasts or commonly used immortalized mouse or rat myoblast cell lines with active 1,25(OH)_2_D_3_. The results of these studies demonstrate positive effects on myoblast differentiation, fusion and myotube formation,^(^ ^9^, ^26^, ^28^, ^29^, ^30^ ^)^ reduced proliferation of early myoblasts in preparation for fusion,^(^ ^29^, ^30^, ^31^ ^)^ and improved myotube maintenance and size.^(^ ^28^ ^)^

Two broad mechanisms are responsible for these pleiotropic effects including: (i) posttranslational modification of signaling molecules involved in inhibition of myoblast proliferation, Rb, JNK, Raf‐1, and CREB^(^ ^9^, ^29^, ^38^ ^)^; and (ii) genomic regulation of myogenic regulatory factors (MRF) responsible for muscle differentiation.^(^ ^9^, ^29^ ^)^ Knockdown studies have confirmed VDR as requisite to the effects of 1,25(OH)D_3_ on MRF expression.^(^ ^39^ ^)^

In vitro studies also report a vitamin D–protective effect against muscle atrophy via the downregulation of muscle atrophy F‐box (MAFbx/Atrogin 1) and muscle RING finger 1 (MuRF1) proteins as well as inhibition of ubiquitin ligases involved in catabolism and muscle atrophy.^(^ ^32^ ^)^

Corroborative findings in studies on human myocytes were reported with VDR‐mediated modulation of age‐related pathways including ubiquitin ligases, inflammatory markers tumor necrosis factor α (TNF‐α) and interleukin 6 (IL6), and phosphatidylinositol‐3′‐kinase (PI3K)/protein kinase B (AKT) signaling.^(^ ^21^, ^40^ ^)^ Mitochondrial gene modulation and increased mitochondrial volume and oxygen consumption in muscle cells treated with 1,25(OH)D_3_ were demonstrated.^(^ ^41^ ^)^ Thus, vitamin D may reduce oxygen free radicals in aging muscle and mitigate the effects of mitochondrial dysfunction, thereby counteracting sarcopenia.

Studies also report increases in intracellular calcium movement in muscle in response to vitamin D.^(^ ^6^ ^)^ In cell models, 1,25(OH)_2_D_3_ exposure leads to the rapid movement of calcium from the sarcoplasmic reticulum into cytosol,^(^ ^42^ ^)^ followed by more sustained calcium flux from the extracellular compartment via activation of store‐operated calcium entry (SOCE) and L‐type voltage‐dependent channels.^(^ ^6^ ^)^ Intricate intracellular signaling mechanisms for these effects have been elucidated, including the activation of the protein kinase C pathway^(^ ^42^ ^)^ and increases in intracellular cyclic AMP (cAMP).^(^ ^43^ ^)^ Interestingly, one study reported the expression of CYP27B1, suggesting local muscle metabolism of 25(OH)D_3_ into 1,25(OH)_2_D_3_.^(^ ^31^ ^)^

Thus, a body of in vitro work provides proof of concept that vitamin D modulates myogenesis, through the inhibition of myoblast proliferation, promotion of myocyte differentiation and myotube formation, and by exerting anabolic effect on myotube size.^(^ ^9^, ^28^, ^29^, ^38^, ^44^ ^)^ Vitamin D also modulates the response to muscle injury and inflammation, with effects on intracellular calcium handling.

## Insights on Vitamin D's Role in Muscle From Animal Models

A number of different animal models have shed light on key aspects of vitamin D physiology and skeletal muscle.

The classical model, namely the whole‐body vitamin D receptor knockout (VDRKO) mouse model has provided key insights in the pleiotropic effects of vitamin D,^(^ ^38^, ^45^ ^)^ but the systemic defects in this model, and aberrant calcium/mineral signaling, confound the elucidation of tissue specific effects of the VDR. More recently, two muscle‐specific knockout mouse models have been generated.^(^ ^46^, ^47^ ^)^ In the first model, *myosin light chain 1* (*MLC1f*) was the promoter gene used to ablate *VDR* in the muscle of these mice. *MLC1f* is expressed in embryonic life, making this model appropriate to assess effects on muscle differentiation. Changes in muscle morphology and insulin sensitivity were noted, with a reduction in type II muscle fiber diameter and overexpression of forkhead Box O1 (FOXO1) protein resulting in glucose intolerance.^(^ ^46^ ^)^ In the second model, significant reductions in lean mass, voluntary physical function, and grip strength in muscle‐specific VDRKO mice, underpinned by key morphologic changes in muscle fibers, were demonstrated.^(^ ^47^ ^)^ The presence of central nucleoli in muscle fibers of knockout mice suggested an underlying defect in muscle repair in the absence of VDR.

A model overexpressing VDR in the tibialis anterior muscle of Wistar rats was recently developed.^(^ ^48^ ^)^ Significant increases in muscle fiber size were reported with upregulation of key genomic pathways on RNA‐Seq relating to extracellular matrix (ECM) remodeling, satellite cell activity, and markers of proliferation.^(^ ^48^ ^)^ In addition, humans were found to upregulate VDR mRNA in skeletal muscle following hypertrophy‐inducing exercise, suggesting a central role in muscle conditioning to exercise and recovery.^(^ ^48^ ^)^

Animal models of vitamin D deficiency have also been developed. Vitamin D–deficient mice had reduced locomotor ability and by 5 weeks on a diet lacking vitamin D, mice had significantly lower neuromuscular innervation in the tibialis anterior muscle compared to calcium and vitamin D–replete animals.^(^ ^49^ ^)^ Muscle atrophy was accentuated by vitamin D deficiency in aged mice with an increase in muscle protein catabolism via activation of TGF‐β, FOXO, and the ubiquitin‐proteasome system.^(^ ^45^, ^50^ ^)^ Conversely, vitamin D supplementation improved muscle recovery following freeze‐crush injury or high‐intensity exercise.^(^ ^51^, ^52^ ^)^ These effects were explained by vitamin D–mediated reductions in oxidative stress and inflammation, together with an effect on stress‐related proteins (ERK1/2, p38, and MAPK).^(^ ^51^, ^53^ ^)^

Murine models of energy dysmetabolism have also been used to investigate the role of vitamin D in muscle health; the prospective muscle atrophy, increased lipid accumulation within skeletal muscle, and overall reduced muscle structure and function render these models useful for studying muscle maintenance, function, and form. Overall, these investigations report that vitamin D treatment in animals fed high‐fat and high‐sugar diets, diabetic rodent models, and sedentary fatty rats preserved skeletal muscle form and function by: increasing myoblast determination protein 1 (MyoD) expression^(^ ^54^ ^)^; suppressing the expression of catabolic proteins atrogin‐1 and MuRF1^(^ ^54^ ^)^; upregulating the expression of protein fibronectin‐type III domain‐containing 5 (FNDC5) and irisin—genes involved in the conversion of white adipocytes into brown adipocytes and increasing energy expenditure^(^ ^55^ ^)^; and the downregulation of the lipogenic activation pathway that includes sterol regulatory element binding protein‐1c (SREBP1c) and SREBP cleavage activating protein (SCAP).^(^ ^56^ ^)^

Despite these positive findings, one study reported that dosing mice with extremely high levels of vitamin D in a single bolus treatment resulted in reduced contraction force and reduced recovery periods following fatigue exercises,^(^ ^57^ ^)^ supporting data from humans in which comparable single high doses increase the risk of falls and fractures.^(^ ^58^ ^)^

These animal studies have laid the foundation for further examination of vitamin D effects in human muscle tissue. A correlation between VDR protein and interleukin‐6 (IL6) expression in human muscle suggests integrated effects in the inflammatory response to muscle damage.^(^ ^59^ ^)^ Vitamin D supplementation modulated cytokine levels following exercise, including IL‐10, IL‐13, and reduced inflammatory mediators TNF‐α and interferon γ (IFN‐γ).^(^ ^60^, ^61^ ^)^ PCR array analysis in healthy human subjects identified 24 skeletal muscle genes associated with pathways involving muscle contraction, myogenesis, cell stress, and muscle repair that correlate with serum 25(OH)D_3_ status.^(^ ^62^ ^)^

In summary, vitamin D modulates muscle morphology and pleoitropy with effects on muscle size, repair, and aging. Effects on oxidative stress, inflammatory cytokines, and muscle protein turnover via the ubiquitin‐proteasome have been reported in both rodent and human studies. Vitamin D supplementation may reverse these effects, and further research is needed on the potential anti‐aging, anabolic, and reparative effects of vitamin D on skeletal muscle.

## Muscle as a Storage Site for Vitamin D

Other recent research has asked not what vitamin D can do for muscle but what muscle can do for vitamin D. At somewhere between 50 to 100 days,^(^ ^63^, ^64^ ^)^ 25(OH)D_3_ has an unusually long half‐life for a steroid hormone, particularly a seco‐steroid with a broken carbon ring, whereas the half‐life of 1,25(OH)_2_D_3_ is more similar to other steroid hormones, at only a few days.^(^ ^65^ ^)^ The half‐life of the vitamin D binding protein (VDBP), which transports both the endocrine metabolite and its parent molecule, is also only a few days.^(^ ^66^ ^)^ These characteristics of 1,25(OH)_2_D_3_, alongside the well‐known dependence on UVB exposure, and thus seasonal variation for the synthesis of vitamin D, make a reasonable argument for an extravascular storage site for vitamin D within the body.^(^ ^67^ ^)^ Given the lipophilic nature of free vitamin D and that it is purportedly sequestered in the fat mass of obese patients, it is unlikely that the vitamin D in adipose can be mobilized and released as required into the circulation.

In cell culture, radiolabeled 25(OH)D_3_ is taken up into mature myotubes but not mature adipocytes.^(^ ^68^ ^)^ The levels in liver are very low,^(^ ^69^ ^)^ and in rats, radioactively labeled vitamin D was found mostly as 25(OH)D_3_ in the skeletal muscle of newborn pups when it had been administered to the pregnant mothers.^(^ ^69^ ^)^ Therefore, muscle appears to be a newly recognized site of accessible 25(OH)D storage.

Although 25(OH)D_3_ can passage into and out of muscle cells unaided, the binding protein, VDBP has been shown to depend on the presence of megalin, a member of the low‐density lipoprotein receptor family.^(^ ^70^ ^)^ VDBP was shown to bind to intracellular skeletal muscle actin,^(^ ^71^ ^)^ in the same manner as had been reported in hepatocytes.^(^ ^71^ ^)^ Thus, it is likely that vitamin D is stored in muscle by actin‐bound VDBP that is proteolyzed to allow release of free 25(OH)D_3_ back into the circulation when UVB exposure is low. Finally, uptake of 25(OH)D_3_ into muscle is inhibited by parathyroid hormone (PTH) acting on PTH receptors in the sarcolemma,^(^ ^72^ ^)^ indicating the interplay between these calciotropic hormones to maintain calcium homeostasis even at the skeletal muscle. Because PTH is a major inducer of CYP27B1 in the kidneys, it is conceivable that during low calcium situations PTH directs 25(OH)D_3_ away from muscle storage to promote renal conversion of the CYP27B1 substrate to 1,25(OH)_2_D_3_. Thus, skeletal muscle represents an active storage site for vitamin D, playing a novel role in the maintenance of physiological circulating levels during periods of hibernation and low UVB exposure.

## Conclusion

In his 2006 review on the “already busy receptor,” the late Anthony Norman described skeletal muscle as a new frontier in VDR's assignments beyond its genomic effects at classical sites.^(^ ^73^ ^)^ This special issue article in memory of this pioneer of vitamin D research summarizes the current understanding of its effects on skeletal muscle—in development, metabolism, repair, and the modulation of muscle fiber size. A body of preclinical research in support of these functions has been presented from in vitro cell models, animal models, and biomolecular studies on human muscle tissue (summarized in Fig. 1).

Morphologically, muscle mass, fiber size, and the reparative response to muscle injury are altered by vitamin D.^(^ ^25^, ^45^ ^)^ Genomic effects in muscle involve myogenic regulatory factors, transforming growth factor β (TGF‐β) signaling, myostatin, and the ubiquitin‐proteasome.^(^ ^9^, ^29^, ^62^ ^)^ Vitamin D may also exert age‐related changes in skeletal muscle by altering oxidative stress, atrophy signaling, and protein turnover.^(^ ^45^, ^50^ ^)^ Nongenomic effects of vitamin D have been elucidated, including the rapid activation of second messenger systems to modulate intramuscular calcium flux, vital for muscle contraction and strength.^(^ ^43^, ^74^, ^75^ ^)^ Skeletal muscle may also represent a dynamic depot for vitamin D, regulating circulating levels and facilitating the release of vitamin D during periods of low UVB exposure such as hibernation.^(^ ^70^ ^)^

Until recently, VDR's expression in skeletal muscle has been controversial. Technical challenges, differences in muscle models, and muscle cell‐specific and age‐specific differences in VDR expression gave rise to this controversy. The body of evidence now indicates that VDR *is* indeed expressed in muscle, but at levels that may elude some detection methods. The expression of VDR in muscle occurs primarily in primordial muscle cells, such as satellite cells, and in regenerating muscle fibers.^(^ ^16^, ^17^ ^)^ VDR's predominant expression in these early muscle cells indicates a mostly pleiotropic role in this tissue. At a functional level, a direct action of VDR in muscle is supported by the phenotype of murine models of muscle‐specific knockout^(^ ^47^ ^)^ and overexpression of this protein.^(^ ^48^ ^)^

However, questions regarding the activity of vitamin D in muscle remain. Although preclinical studies support a robust role of vitamin D in this tissue, the clinical translation of these effects to human subjects requires further assessment. Direct changes in muscle function relating to vitamin D status have not been a uniform finding in human studies. On a molecular level, the presence of vitamin D response elements (VDRE) have not been clearly demonstrated in genes purportedly regulated by vitamin D in muscle. Chromatin immunoprecipitation (ChIP) studies are required to elucidate the specific VDR binding sites across the genome (or cistrome) in skeletal muscle and will establish a clearer picture of direct genomic activity. Nongenomic effects of vitamin D on calcium flux have been reported by many in vitro studies but the extrapolation of these findings to in vivo muscle physiology is not a *fait accompli*. Future studies using intracellular calcium imaging are needed to characterize real‐time calcium flux in skeletal muscle in vivo in response to vitamin D. The role of Vitamin D in muscle repair also raises the possibility that vitamin D directly regulates the response of satellite cells to injury, enhancing regeneration. The ongoing development of animal models of aberrant VDR signaling in muscle will help to clarify cell‐specific effects in this tissue.

Future strategies targeting vitamin D signaling in muscle and modulating the VDR “metabolome” may also hold important clues for future treatments of musculoskeletal disorders, congenital myopathies, and sarcopenia.

## Conflict of Interest

The authors have no conflict of interest to report.

### PEER REVIEW

The peer review history for this article is available at https://publons.com/publon/10.1002/jbm4.10575.
